# A Retrospective Linked Data Analysis of Acute Rheumatic Fever and Rheumatic Heart Disease Diagnoses in Children Aged Under Five Years in Australia, 2001–2017

**DOI:** 10.1111/ajr.70135

**Published:** 2026-01-14

**Authors:** Jamie Cransberg, Judith Katzenellenbogen, Bo Remenyi, Carl Francia, Kevin Murray, Ingrid Stacey

**Affiliations:** ^1^ Cardiovascular Epidemiology Research Centre, School of Population and Global Health University of Western Australia Perth Western Australia Australia; ^2^ Charles Darwin University, Menzies School of Health Research Darwin Northern Territory Australia; ^3^ University of Queensland, School of Health and Rehabilitation Sciences Brisbane Queensland Australia; ^4^ Victor Chang Cardiac Research Institute, Cardiology Population Health Laboratory Perth Western Australia Australia

**Keywords:** aboriginal and Torres Strait islander, acute rheumatic fever, linked data, paediatric cardiology, rheumatic heart disease

## Abstract

**Objective:**

To describe the clinical and demographic profile of Australian children first diagnosed with acute rheumatic fever (ARF) or rheumatic heart disease (RHD) before the age of 5 years, with comparison to children aged 5–14 years.

**Methods:**

Linked emergency department, hospitalisation, RHD register and death records from the End RHD in Australia: Study of Epidemiology were used to identify first ARF/RHD diagnosis occurring in < 15‐year‐olds. Demographic/clinical profiles and pre‐diagnosis healthcare interactions were analysed with stratification into 5‐year age groups.

**Design:**

Retrospective cross‐sectional linked administrative data analysis.

**Setting:**

Northern Territory, South Australia, Queensland and Western Australia.

**Participants:**

Children aged < 15 years at first hospitalisation or notification for ARF or RHD, 2001–2017.

**Main Outcome Measures:**

Disease stage and severity at diagnosis, register notification status, clinical history prior to ARF or RHD diagnosis.

**Results:**

Of 2382 children diagnosed with ARF/RHD aged < 15 years, 180 (7.6%) were aged under 5 years. Among under 5‐year‐olds with ARF or RHD, 30.6% had not been notified to RHD registers. A total 49 under 5‐year‐olds were diagnosed with RHD; with 22 (44.9%) classified as having mild disease, 16 (32.7%) moderate and 6 (12.2%) severe. High hospitalisation rates for injury in the first year of life were observed for the < 5‐year‐old cohort with ARF/RHD.

**Conclusions:**

We present the first comprehensive Australian evidence that ARF and RHD diagnoses are occurring in Australian children aged under 5 years. Greater awareness among clinicians is needed regarding ARF/RHD as a potential diagnosis in this young, high risk age group.

## Abstract (highlights)

**What This Paper Adds:**

- Linked administrative health data was used to describe acute rheumatic fever (ARF) or rheumatic heart disease (RHD) cases among preschoolers.
- ARF/RHD was diagnosed among 180 children aged under 5 years in Australia, 2001–2017.
- Greater clinician awareness about the diagnosis and notification of ARF and RHD within this young age group is needed.

**What Is Already Known on This Subject:**

- There are no comprehensive Australian studies of ARF or RHD in under 5‐year‐olds.
- An analysis of 23 cases of ARF identified in under 7‐year‐olds from the National Child Development Study in the United Kingdom identified history of infection, large household size and migration as ARF risk factors in this age group.

## 1 Introduction

Acute rheumatic fever (ARF) and rheumatic heart disease (RHD) are preventable sequelae of Strep‐A infection [1]. Acute rheumatic fever (ARF) and RHD are driven by social determinants of health including access to healthcare, education, and household environments [1, 2]. There are an estimated 33.5 million prevalent cases of RHD globally, associated with over 285 000 annual deaths, and significant morbidity [3]. In Australia, Aboriginal and Torres Strait Islander peoples (hereafter respectfully ‘Indigenous’) experience an inequitably high burden of ARF and RHD [4].

In 2020, the End RHD in Australia: Study of Epidemiology (ERASE) provided contemporary estimates of Australian ARF and RHD burden using linked administrative health data from RHD registers and other sources [4, 5]. End RHD in Australia: Study of Epidemiology (ERASE) identified that ARF and RHD age‐specific incidence was highest in children aged 5–14 years, and, in line with international estimates, [6, 7]. Acute rheumatic fever (ARF) incidence peaked at 11 years of age [4]. Concerns have been raised about ARF and/or RHD diagnosed in children aged under 5 years in Australia (Bo Remenyi, personal communication September 2021), which is younger than the commonly cited 5–14 year ‘peak’ age of diagnosis. The consequences of RHD in remote Australia have been described in the media ‘children with severe rheumatic heart disease usually die suddenly and unexpectedly children as young as 4 years of age have passed away from rheumatic heart disease’ [8].

Information regarding ARF and RHD epidemiology and associated risk factors specific to children aged under 5 years is scarce, both globally and within Australia. International studies have suggested children with ARF who are aged under 5 years’ experience more severe carditis than children presenting at older ages and have higher rates of familial RHD [9, 10]. Cannon et al. examined 23 historical cases of ARF aged under 7 years at diagnosis in the United Kingdom, identifying history of infections, large households and migration as ARF risk factors in this age group [11]. Additionally, younger age at first ARF episode has been associated with increased risk of RHD development (2% lower risk of RHD per 1 year increase in age at ARF diagnosis) [12]. The RHD Endgame strategy, which outlines a roadmap for eliminating RHD in Australia by 2031, suggests that reduction of minor skin trauma (micro‐abrasions) as a strategy to decrease ARF/RHD disease burden by reducing the risk of skin Strep‐A infections [2]. There are presently no comprehensive Australia wide studies that focus on ARF/RHD diagnosis in children aged under 5 years.

To address this gap, this study describes the frequency and characteristics of Australian children first diagnosed with ARF or RHD aged under 5 years and compares this to characteristics of children at the same age, whose ARF/RHD diagnosis occurred later, aged 5–14 years. First, we describe the demographic profile of children under 5 years and aged 5–14 years diagnosed with first‐ever ARF or RHD in NT, South Australia (SA), Queensland (Qld) and Western Australia (WA) during 2001–2017. Secondly, we describe the clinical and historical profile for the subset of children with complete data coverage since birth and report hospital admission rates for infection and injury that preceded diagnosis.

## 2 Methods

### 2.1 Study Design and Data Sources

This was a cross‐sectional analysis of linked Emergency Department (ED), hospitalisation, RHD register and death data from the ERASE [5]. ERASE is comprised of linked administrative health data from NT, WA, QLD, SA and New South Wales (NSW). Hospital, RHD register or death records with any diagnosis of ARF or RHD between 2001 and 2017 define the ERASE cohort; any corresponding ED, hospital, RHD register and death records for these people regardless of reason are also available to provide comprehensive demographic and clinical information for the cohort during the study period [5].

### 2.2 Sample Selection

The sample for the first objective (demographic profile) included individuals from SA, NT, Qld or WA in the ERASE cohort first diagnosed with ARF or RHD and aged < 15 years between 1 July 2001 to 31 December 2017 (Appendix A). New South Wales (NSW) residents were excluded, as ERASE lacks NSW RHD register information (as RHD register notifications commenced in late 2015 for this jurisdiction). First‐ever ARF or RHD diagnosis date, and corresponding age at diagnosis, were identified across all ERASE data sources. A lookback period was required for hospital‐only identified cases to ensure the first diagnosis within the study period was the first probable diagnosis within a lifetime. As such, hospital‐only cases were included from age 3 years at commencement of the study period, minimising the risk of previous first diagnosis. Register cases have a clinically confirmed date of first diagnosis and thus do not require a lookback period. Consequently, register‐identified cases first diagnosed < 15 years and born after 1 July 1986 and hospital‐identified cases born after 1 July 1998 were included.

For the second objective (clinical and historical profile), the sample described in the first objective was restricted to cases born after 1 July 2001, allowing all hospital records since birth to be reviewed.

### 2.3 Variable Definitions

#### 2.3.1 Acute Rheumatic Fever (ARF) and Rheumatic Heart Disease (RHD) Diagnoses

Individuals identified from jurisdictional registers were denoted ‘register cases’, whereas cases captured only within hospital admission data were defined as ‘hospital‐only cases’. ARF/RHD cases that appeared on registers were considered clinically confirmed. Cases identified from inpatient hospitalisation records were selected based on International Statistical Classification of Diseases and Related Health Problems; Australian Modification (ICD‐10‐AM)‐coded diagnoses [13]. ARF was defined as a primary diagnosis code for ARF (ICD‐10‐AM codes I00‐I02). For RHD, a validated algorithm was applied to hospitalisation records to identify probable RHD cases (ICD‐10‐AM codes I05‐I09, any diagnosis field) to reduce known misclassification and over‐ascertainment associated with ICD‐10‐AM RHD codes [14, 15]. The date of first ARF or RHD diagnosis was assigned according to earliest ARF or RHD date from either data source. The sample was stratified into three age groups based on age at first ARF or RHD diagnosis: 0–4, 5–9, and 10–14 years.

#### 2.3.2 Demographic Characteristics

Sex was assigned using the most frequent value across all records. Population group was defined as Indigenous, ILIC, or other Australian; this classification was based on Indigenous identifiers in hospital/ED records, register‐recorded ethnicity or World Bank income classification of country of birth in hospital data as previously described [16]. Jurisdiction denotes which state‐based register program or healthcare system the ARF/RHD record was sourced from, with linked SA cases [5] classified as NT cases. Geographical variables including remoteness, region and socio‐economic indexes for areas (SEIFA), were assigned based on 2006 area‐level SA2 concordances (obtained by Australian Bureau of Statistics [ABS]) [17]. Region was based on aggregation of Indigenous regions into nine groups (Appendix B) [18]. Remoteness was classified using the Accessibility/Remoteness Index of Australia (ARIA) [19]. Remoteness was manually assigned for 164 people, based on register data about the geographical location of the health provider. Socio‐economic status was assigned using the SEIFA index of relative socio‐economic disadvantage, expressed as quintiles from least to most disadvantaged.

#### 2.3.3 Clinical Characteristics

Health care episodes prior to ARF/RHD diagnosis and features of the diagnostic episode were assessed. First diagnoses were classified as ARF, RHD or concurrent (ARF and RHD within 90 days). Time from diagnosis to ARF/RHD register notification was calculated and divided into the following six categories: missing (notified, no date recorded), registration preceding diagnosis, immediate (within 2 days), non‐immediate (within 30 days), delayed (within 6 months), and never notified (hospital‐only cases). For registered RHD diagnoses, severity at diagnosis was assigned based on notification information, specialist visits and echocardiograms as per the 2020 Australian guideline for prevention, diagnosis and management of ARF and RHD (hereafter ‘National Guidelines’) [20]. RHD severity closest to the diagnosis date was selected; in cases of multiple records, highest severity rating was assigned. Emergency department (ED) presentations 3 months prior to first ARF/RHD diagnosis were identified for each individual. ICD10‐AM primary and additional diagnosis codes from hospital admissions data were investigated and codes for confirmed or possible bacterial infections flagged (Appendix C). These codes were stratified into seven infection types: lower respiratory, skin, upper respiratory, ear, perinatal, Strep‐A, and other. Broad ICD‐10‐AM codes for injury were also defined and admissions containing these codes in primary or additional diagnoses were identified.

### 2.4 Analysis

Demographic summary statistics were stratified by 5‐year age group, with frequencies and proportions presented. For clinical features, frequencies, proportions and rates per 100 person‐years with 95% confidence intervals were calculated. Observed proportions within strata were compared to overall age distribution of cases using a chi‐squared goodness of fit test (*G* test).

Admission rates for infection and injury in the first year of life were calculated, per 100 person‐years. Number of admissions contributed to numerators and person‐years in study formed denominators within each age group.

Analyses were conducted using Statistical Package for the Social Sciences (SPSS) version 28.0.1.0, SAS v9.4 and R version 4.4.1.

### 2.5 Ethics Approvals

The following Aboriginal Ethics Committees approved this study: Western Australian Aboriginal Health Ethics Committee (Project Reference number: 717) and The Aboriginal Health Research Ethics Committee of the Aboriginal Health Council of South Australia Inc. (Reference number:04‐16‐700). Approvals were also received from the Human Research Ethics Committees (HREC) within Menzies School of Health Research (HREC of the Northern Territory Department of Health and Menzies School of Health Research No. 2016–2705, incorporating an Aboriginal sub‐committee) and the Health Departments of Western Australia (Department of Health WA HREC No. 2016/29), South Australia (South Australian Department for Health and Ageing HREC No. HREC/16/SAH/120) and Queensland (Metro North Hospital and Health Service—The Prince Charles Hospital HREC No. HREC/15/QPCH/289). Ethics for this project were approved with a ‘waiver of consent’.

## 3 Results

### 3.1 Demographic Characteristics

Of 2382 individuals in the objective one sample of under 15‐year‐olds with first‐ever ARF or RHD, 180 (7.6%) were first diagnosed at under 5 years of age (Table 1). The age ranges were 1–4 years for ARF and 2–4 years for RHD diagnoses. Within the sample, 91.0% (2166/2382) of cases were notified to state‐based RHD registers, however, the proportion of cases notified to registers was 69.4% (125/180) among under 5‐year‐olds with ARF or RHD. Under 5‐year‐olds with ARF or RHD were frequently Indigenous (87.2%, 157/180), from very remote regions (50.6%, 91/180) or from the most disadvantaged SEIFA quintile (66.7%, 120/180). Northernmost Qld and NT regions of Australia had the highest frequency of under 5‐year‐old ARF and RHD cases; 62 and 51 respectively.

**TABLE 1:** Baseline characteristics of the study sample selected for demographic profiling, stratified by age at first diagnosis with acute rheumatic fever or rheumatic heart disease.

		0‐4 years		5‐9 years		10‐14 years		Total	
		n	%	n	%	n	%	n	%
Total		180		955		1247		2382	
case ascertainment	Hospital records only	55	(30.6)	98	(10.3)	63	(5.1)	216	(9.1)
	Register notified case	125	(69.4)	857	(89.7)	1184	(94.9)	2166	(90.9)
Sex	Male	99	(55.0)	486	(50.9)	629	(50.4)	1214	(51)
	Female	81	(45.0)	469	(49.1)	618	(49.6)	1168	(49)
Population group									
	Indigenous	157	(87.2)	880	(92.1)	1151	(92.3)	2188	(91.9)
	ILIC	< 5	—	41	(4.3)	59	(4.7)	103	(4.3)
	Other	20	(11.1)	34	(3.6)	37	(3.0)	91	(3.8)
Remoteness area									
	Missing/no fixed address	< 5	—	17	(1.8)	35	(2.8)	53	(2.2)
	Metro and inner regional	14	(7.8)	68	(7.1)	104	(8.3)	186	(7.8)
	Outer regional	37	(20.6)	173	(18.1)	223	(17.9)	433	(18.2)
	Remote	37	(20.6)	164	(17.2)	224	(18.0)	425	(17.8)
	Very remote	91	(50.6)	533	(55.8)	661	(53)	1285	(53.9)
Jurisdiction									
	SA/NT	71	(39.4)	433	(45.3)	589	(47.2)	1093	(45.9)
	QLD	79	(43.9)	368	(38.5)	465	(37.3)	912	(38.3)
	WA	30	(16.7)	154	(16.1)	193	(15.5)	377	(15.8)
Region									
	Any metro region	16	(8.9)	62	(6.5)	91	(7.3)	169	(7.1)
	NSW non‐metro	< 5	—	< 5	—	< 5	—	< 5	—
	QLD North	62	(34.4)	299	(31.3)	370	(29.7)	731	(30.7)
	QLD non‐metro	6	(3.3)	25	(2.6)	24	(1.9)	55	(2.3)
	SA non‐metro	< 5		< 5	—	< 5	—	< 5	—
	WA North	18	(10.0)	103	(10.8)	125	(10.0)	246	(10.3)
	WA non‐Metro	8	(4.4)	34	(3.6)	35	(2.8)	77	(3.2)
	NT Central	18	(10.0)	113	(11.8)	135	(10.8)	266	(11.2)
	NT Top End	51	(28.3)	301	(31.5)	435	(34.9)	787	(21.0)
	Not available	< 5	—	14	(1.5)	29	(2.3)	44	(1.8)
SEIFA Quintiles (least to most disadvantaged)									
	Missing	14	(7.8)	92	(9.6)	182	(14.6)	288	(12.1)
	1 (least disadvantaged)	< 5	—	6	(0.6)	19	(1.5)	26	(1.1)
	2	9	(5.0)	38	(4.0)	44	(3.5)	91	(3.8)
	3	15	(8.3)	52	(5.4)	86	(6.9)	153	(6.4)
	4	21	(11.7)	86	(9.0)	120	(9.6)	227	(9.5)
	5 (most disadvantaged)	120	(66.7)	681	(71.3)	796	(63.8)	1597	(67)

Abbreviations: ILIC, immigrant from low‐ and middle‐ income country; Metro, metropolitan; NT, Northern Territory; Qld, Queensland; SA, South Australia; SEIFA, socio‐economic indexes for areas; WA, Western Australia.

### 3.2 Clinical Characteristics

#### 3.2.1 At Diagnosis

Among 1209 individuals included in the objective two sample (i.e., children with complete ERASE data coverage available since birth), 158 (13.0%) were aged under 5 years (Table 2). Most under 5‐year‐olds were first diagnosed with ARF (117/158, 74.1%), however 15 were diagnosed with RHD and 26 were diagnosed with concurrent ARF and RHD (representing 9.5% and 16.5% respectively). Immediate RHD register notification was done for 81/158 (51.3%) under 5‐year‐olds; notification was delayed more than 1 month for 5/158 (3.2%) and not done in 49/158 (31.0%). By contrast, 930/1051 (88.5%) of ARF/RHD cases diagnosed aged 5–14 were notified. Among the total 49 RHD diagnoses in under 5‐year‐olds, 22 (44.9%) were classified as having mild disease, 16 (32.7%) were moderate and 6 (12.2%) were severe. Although > 80% of under 5‐year‐olds had no recorded ED presentation in the 3 months prior to their first ARF or RHD diagnosis, 29 had attended ED at least once.

**TABLE 2:** Clinical features of children with acute rheumatic fever (ARF) or rheumatic heart disease (RHD) that had complete data coverage since birth, stratified by age group at ARF/RHD diagnosis.

		0–4 years		5–9 years		10–14 years		Total		
		n	(%)	n	(%)	n	(%)	n	(%)	*p* value^c^
Total		158		643		408		1209		
Disease stage at first diagnosis	ARF	117	(74.1)	420	(65.3)	250	(61.3)	787	(65.1)	0.239
	RHD^a^	15^a^	(9.5)	58	(9.0)	39	(9.6)	112	(9.3)	0.957
	Concurrent ARF/RHD	26	(16.5)	165	(25.7)	119	(29.2)	310	(25.6)	0.027
Time to RHD register notification	Missing (notified, unknown date)	10	(6.3)	39	(6.1)	32	(7.8)	81	(6.7)	0.545
	Registration prior to diagnosis	< 5	—	5	(0.8)	< 5	—	6	(0.5)	—
	Immediate (within 2 days)	81	(51.3)	424	(65.9)	294	(72.1)	799	(66.1)	0.024
	Non‐immediate (within 30 days)	12	(7.6)	77	(12.0)	38	(9.3)	127	(10.5)	0.207
	Delayed (within 6 months)	5	(3.2)	17	(2.6)	< 5	—	26	(2.2)	—
	Never Notified (hospital‐only)	49	(31.0)	81	(12.6)	40	(9.8)	170	(14.1)	< 0.001
RHD severity at diagnosis (Register RHD diagnoses only)	Total RHD diagnosis	49		275		174		498		
	Missing	5	(10.2)	20	(7.3)	< 5	—	27	(5.4)	—
	Severe	6	(12.2)	33	(12.0)	19	(10.9)	58	(11.6)	0.780
	Moderate	16	(32.7)	64	(23.3)	41	(23.6)	121	(24.3)	0.998
	Mild	22	(44.9)	158	(57.5)	110	(63.2)	290	(58.2)	0.016
	Other^b^	< 5	—	< 5	—	< 5	—	< 5	—	—
ED presentation (all cause)^d^ 3‐months prior to diagnosis	2 or more	16	(10.1)	29	(4.5)	14	(3.4)	59	(4.9)	0.004
	1	13	(8.2)	92	(14.3)	53	(13.0)	158	(13.1)	0.166
	0	129	(81.6)	522	(81.2)	341	(83.6)	992	(82.0)	0.915

Abbreviations: ARF, acute rheumatic fever; ED, emergency department; RHD, rheumatic heart disease.

^a^ The frequency of total RHD diagnoses reported is larger than that obtained by adding first diagnosis of RHD with concurrent ARF/RHD due to disease progression within the age category during the study period. For example, *n* = 8 ARF episodes among < 5‐year‐olds subsequently progressed to RHD before the age of 5 years, making total RHD diagnoses *n* = 49 (and not *n* = 41).

^b^ Includes borderline and inactive disease.

^c^ Chi‐square goodness of fit test to compare observed distribution to overall age distribution (*G* test).

^d^ N.B. ‘ED presentations’ are dependent upon local access to an ED department or by air/road retrieval.

Compared to 5–14‐year‐olds with ARF or RHD, under 5‐year‐olds were diagnosed with concurrent ARF/RHD less frequently (compare concurrent ARF/RHD: 25.7% in 5–9‐year‐olds and 29.2% in 10–14‐year olds versus 16.5% in < 5‐year‐olds, *p* = 0.027, Table 2). Under 5‐year‐olds with ARF/RHD were less frequently notified to RHD registers within 2 days of diagnosis and were more frequently never notified compared to older children. When the first diagnosis was RHD rather than ARF or concurrent illness, under 5‐year‐olds were less frequently diagnosed with mild disease than older children (compare 44.9% mild RHD in under 5‐year‐olds to 57.5% 5–9‐year‐olds and 63.2% 10–14 year olds, *p* = 0.016, Table 2).

#### 3.2.2 History of Hospitalisation or ED Presentation in Early Life

Children aged under 5 years with ARF/RHD presented more frequently to ED in the 3 months preceding ARF/RHD diagnosis than older age groups, with two or more presentations in 10.1% of children under 5 years (*p* = 0.004, Table 2 and Appendix D).

**FIGURE 1:**
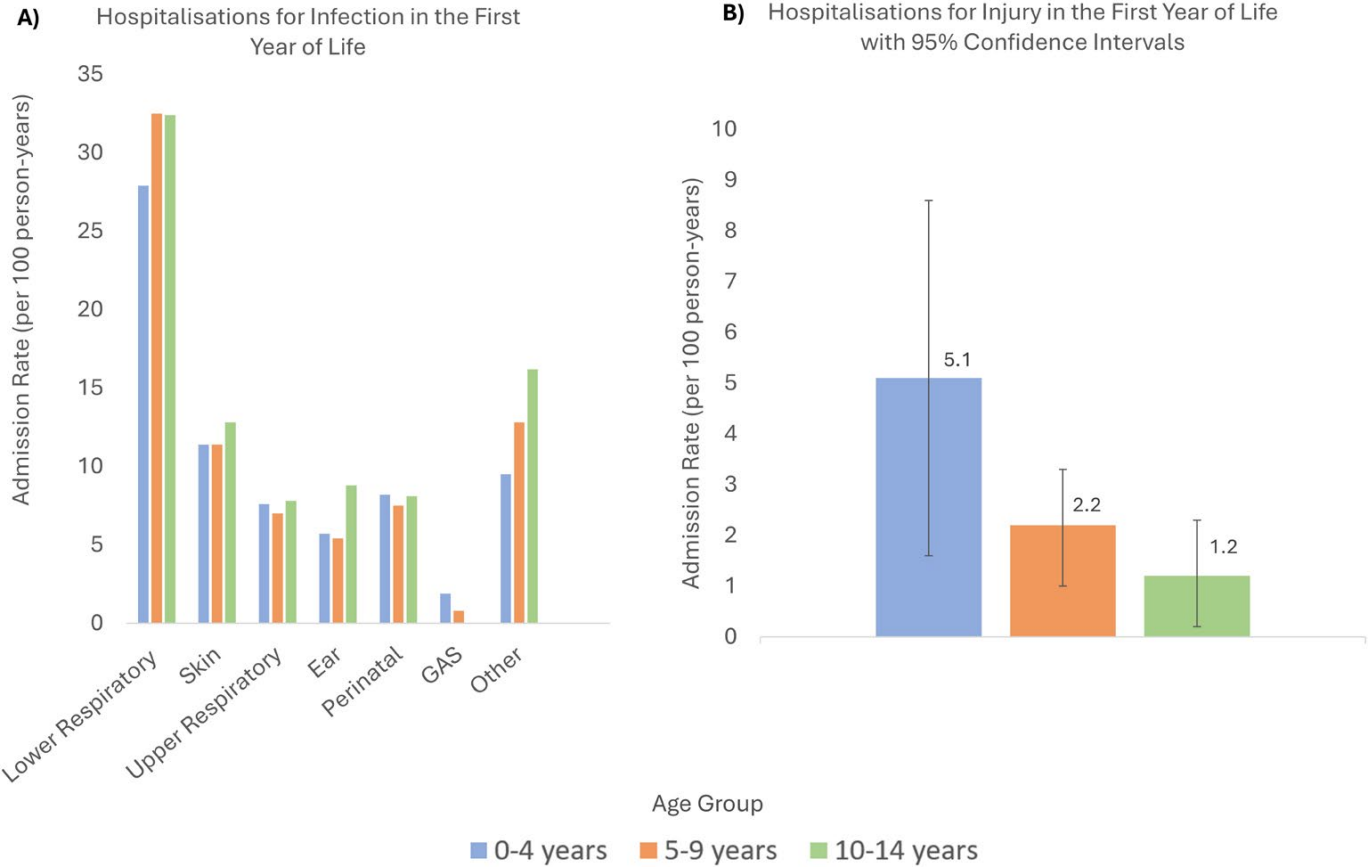
Hospital admission rates per 100 person‐years in the first year of life by age group at ARF/RHD diagnosis for (A) infections and (B) injury (Appendix D).

Children diagnosed with ARF/RHD under 5 years had 72.2 (95% CI, 58.9–85.4) admissions per 100‐person‐years in their first year of life for any infection, which was comparable to rates observed in older children (77.3 [95% CI, 70.5–84.1] in 5–9‐year‐olds and 86.0 [95% CI, 77.0–95.0] in 10–14‐year‐olds). In the first year of life, children with a diagnosis of ARF/RHD under 5 years experienced 1.9 hospitalisations per 100‐person‐years for Strep‐A infections, 7.6 hospitalisations per 100‐person‐years for upper respiratory tract infections and 11.4 hospitalisations per 100‐person‐years for skin infections (Figure 1). Hospitalisation rates in the first year of life were comparable for all < 15‐year‐old age groups for the infection types investigated.

Injury related hospitalisations in under 5‐year‐olds with ARF/RHD occurred at a rate of 5.1 (95% CI, 1.6–8.6) admissions per 100 person‐years in the first year of life (Figure 1), with 2.2 (95% CI, 1.0–3.3) and 1.2 (95% CI, 0.2–2.3) per 100 person years among children aged 5–9 and 10–14 years respectively.

## 4 Discussion

This is the first Australian study that investigated the demographic and clinical characteristics of ARF and RHD cases diagnosed in children under 5 years. Despite perceived rarity, ARF and RHD of varying severity is being diagnosed in Australian children aged under 5 years, with remote‐residing and Indigenous Australian children over‐represented within this young cohort. During 2001–2017, there were 180 cases of ARF or RHD diagnosed in children aged under 5 years in NT, SA, Qld and WA. Concerningly, when RHD was diagnosed in children aged under 5 years it was less frequently classified as mild disease; and children with ARF or RHD aged under 5 years were missing from RHD registers more often than 5–14‐year‐old children, increasing the risk that this cohort will experience poor health outcomes in the future.

Children under 5 years were most frequently first diagnosed with ARF in the absence of RHD, representing an opportunity to prevent recurrences and progression to RHD or further complications via notification to RHD registers for ongoing monitoring and secondary prophylaxis administration. Unfortunately, diagnosis at the ARF stage did not guarantee notification to RHD registers, with 30.6% of children diagnosed under 5 years missing from registers in this study. Although younger children and people with ARF have been reported to be notified to RHD registers at higher rates than older individuals and those with RHD, the under 5‐year‐old cohort in this study was found to represent an under‐notified demographic [21]. It is unclear whether notification of ARF/RHD among under 5‐year‐olds to RHD registers has increased over time; our recent review of medical records in one WA region has indicated that under‐notification of true cases persists [22]. It is possible that lack of diagnostic certainty might influence propensity to notify in under 5‐year‐olds, however our study could not capture these cases. Whilst the real‐world evidence of RHD register program efficacy is still weak there is a general acceptance globally that early diagnosis, regular secondary prophylaxis and the delivery of coordinated care are the best clinical management strategies available where primary prevention has failed [23, 24, 25, 26]. Our findings support the need for improved clinician awareness of ARF and RHD onset in children aged under 5 years and the need for RHD register notification when a diagnosis has been made.

Children diagnosed with ARF or RHD before the age of 5 years were most frequently resident in remote or very remote areas, with the highest numbers of cases observed in the northernmost NT and Qld regions. Northern Australian and remote residents are known to experience the highest burden of ARF and RHD in Australia, so this observation is not unexpected [4]. It is believed that this epidemiology is due to a higher Strep‐A burden in these Australian regions, due to the complex intersection of low access to health care services, the ongoing impacts of colonisation and racism leading to socio‐economic deprivation and climate [27]. Interestingly, we did not observe differences in the prior hospitalisation of under 5‐year‐olds for infectious diseases prior to their first ARF or RHD diagnosis, when compared to the 5–14‐year‐olds. The high number of ARF and RHD cases in under 5‐year‐olds in Northern, remote Australia necessitates a special and urgent focus by service providers and RHD control programs in these regions.

A trend towards higher rates of hospitalised injury in the first year of life (HIY1) among children diagnosed with ARF/RHD in younger age groups was observed in our study. Children diagnosed with ARF or RHD under 5 years of age had the highest HIY1 rates in this study, with 5.1 (95% CI, 1.6–8.6) hospitalisations per 100‐person‐years, which is higher than the background Australian population rate of 0.8 admissions per 100‐person‐years reported by the Australian Institute of Health and Welfare (equivalent methodology) [28]. Strep‐A skin infections and minor skin trauma preceding these infections have been implicated in the development of ARF in Australia and New Zealand [29, 30, 31], and ARF is also documented more generally in environments with high Strep‐A bacterial burdens [32, 33, 34, 35, 36, 37, 38]. Higher injury and trauma‐related HIY1 rates are likely associated with both socio‐economic disadvantage and rural residential location, however, regardless of cause these early life healthcare interactions preceding ARF or RHD diagnosis can be viewed as opportunities to offer health promotion education and facilitate prevention.

## 5 Study Limitations and Strengths

Since ERASE is a linked administrative data collection, it has not been specifically designed for clinical research which limits the availability of data for analysis, especially for individuals who are only found in hospital records and have not been notified to RHD registers. Hospital‐identified RHD cases lack detailed clinical information such as RHD severity. Additionally, ICD‐10‐AM coding is prone to RHD misclassification, however our use of a validated predictive algorithm has reduced the impact of this limitation [14, 15]. Another major limitation is the lack of comprehensive primary care data for these young children, which would provide valuable information about early opportunities for ARF and RHD prevention outside of the hospital and ED setting; unfortunately, this information was not systematically available within the ERASE data collection [5]. We acknowledge that ‘ED presentations’ is a crude metric that simplifies multiple place‐based factors, including access to a staffed local clinic, regional retrieval practises and local childhood injury patterns [39]. Small numbers of observed events (e.g., trauma hospitalisations) have precluded our ability to interrogate findings using multivariable models. This analysis was unable to investigate the true burden of RHD among ILIC groups, given the lack of accurate statistics on migration/ethnicity and potential for missed diagnoses. Despite this, the present study provides the most geographically complete, long‐term description of ARF and RHD in under 5‐year‐old Australian children.

## 6 Conclusion

Using the ERASE linked data collection, we describe 180 cases of ARF or RHD in children under 5 years over 2001–2017 and demonstrate that, contrary to dominant beliefs, both ARF and RHD are indeed occurring in this very young age group. Our findings indicate that many children diagnosed with ARF/RHD before the age of 5 years are not notified to registers, representing a missed opportunity for optimal follow up and potential improvement in outcomes. These children do have other frequent healthcare interactions for infection and injury in their first year of life however, offering opportunity for early intervention. It is imperative that clinicians are aware of this silent disease burden and consider ARF and RHD as differential diagnoses in children under 5 years; and notify all diagnoses to jurisdictional registers. By demonstrating the impacts of remoteness and socio‐economic status across children of all ages, we provide further support for full implementation of the Endgame strategy, a comprehensive strategy to eliminate RHD in Australia.

## Author Contributions

**Jamie Cransberg:** conceptualization, investigation, writing – original draft, methodology, writing – review and editing, formal analysis, visualization. **Judith Katzenellenbogen:** conceptualization, data curation, funding acquisition, supervision, writing – review and editing. **Bo Remenyi:** writing – review and editing. **Carl Francia:** cultural oversight, writing – review and editing. **Kevin Murray:** methodology, supervision, writing – review and editing. **Ingrid Stacey:** writing – review and editing, supervision, data curation, conceptualization, project administration.

## Funding

This study was supported by National Health and Medical Research Council project grant no. 1146525; National Heart Foundation Future Leader Fellowship no. 108106 and Postdoctoral Fellowship no. 110335.

## Ethics Statement

The following Aboriginal Ethics Committees approved this study: Western Australian Aboriginal Health Ethics Committee (Project Reference number: 717) and The Aboriginal Health Research Ethics Committee of the Aboriginal Health Council of South Australia Inc. (Reference number: 04–16‐700). Approvals were also received from the Human Research Ethics Committees (HREC) within Menzies School of Health Research (HREC of the Northern Territory Department of Health and Menzies School of Health Research No. 2016–2705, incorporating an Aboriginal sub‐committee) and the Health Departments of Western Australia (Department of Health WA HREC No. 2016/29), South Australia (South Australian Department for Health and Ageing HREC No. HREC/16/SAH/120) and Queensland (Metro North Hospital and Health Service—The Prince Charles Hospital HREC No. HREC/15/QPCH/289). Ethics for this project were approved with a ‘waiver of consent’.

## Conflicts of Interest

The authors declare no conflicts of interest.

## Supporting information


**Data S1:** Supplementary Information.

## Data Availability

The data that support the findings of this study are available on request from the corresponding author. The data are not publicly available due to privacy or ethical restrictions.
