# Supplementary material for: A Retrospective Linked Data Analysis of Acute Rheumatic Fever and Rheumatic Heart Disease Diagnoses in Children Aged Under Five Years in Australia, 2001–2017
Source: Aust J Rural Health. 2026 Jan 14;34(1):e70135. doi: 10.1111/ajr.70135 (PMC12800885; doi:10.1111/ajr.70135)
Supplement: Supplementary file 1 — Data S1: Supplementary Information. [file AJR-34-0-s001.docx]

**
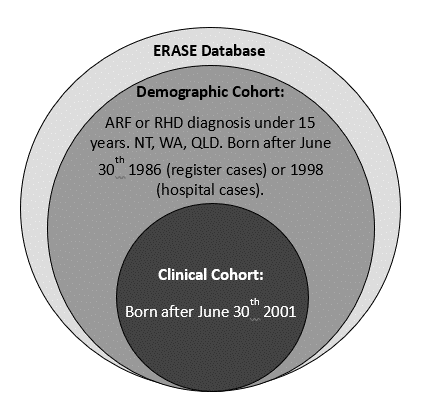
**

**
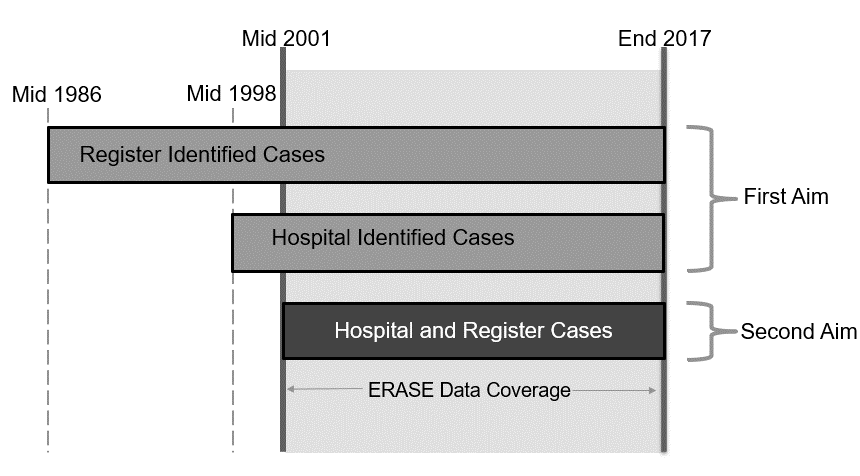
**

## **Appendix A:**

Schematic describing cohort derivation for the first and second research objective and how this relates to the ERASE data coverage period.


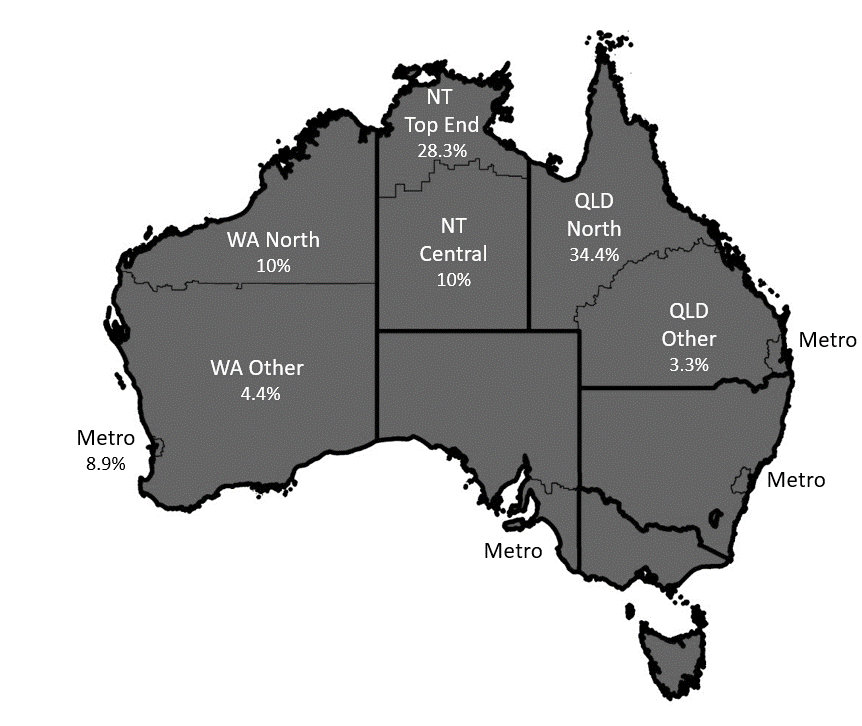


**Appendix B.** Classification of study regions used in the ERASE study, these were generated by combining multiple Indigenous Regions (Australian Bureau of Statistic geographical data structure).

## **Appendix C:**

ICD-10-AM codes utilised in this study are described below. The codes for infections have been adapted from previous work by Baker et al (2012). (1)

Reference: Baker MG, Barnard LT, Kvalsvig A, Verrall A, Zhang J, Keall M, et al. Increasing incidence of serious infectious diseases and inequalities in New Zealand: a national epidemiological study. Lancet. 2012;379(9821):1112-9.

**Table 1.** Summary of ICD-10-AM codes for infection and injury utilised on objective two of this study.

| **Category** | **Codes** |
| --- | --- |
| **Enteric/gastrointestinal Infections** | A00- cholera  A01- typhoid and paratyphoid  A02- salmonella  A03- shigella  A04- E. coli, Campylobacter, yersinia, C. Diff, other unspecified  A05- food-borne  A09.0- unspecified infectious colitis  K35, K36, K37- appendicitis  K61 ano-rectal abscess  K63.0- abscess of intestine  K67- peritonitis  K75.0- liver abscess  K77- other infectious liver disease  K83.0- cholangitis |
| **Lower Respiratory Infections** | A48.1, A48.2- legionnaires disease  B59- pneumocystis  J13- streptococcus pneumonia  J14- pneumonia H influenzae  J15- bacterial pneumonias  J16, J17, J18- pneumonias  J20, J40 -bronchitis  J21- bronchiolitis  J22- unspecified lower respiratory tract infection  J85.3- lung abscess  J86- pyothorax  A15, A16, J65- tuberculosis |
| **Skin Infections** | A46- erysipelas  L00- staph scalded skin  L01- impetigo  L02- abscess  L03- cellulitis  L04- lymphadenitis  L05- pilonidal  L08- other skin infections  B86- scabies  T80.2, T81.4- post operative wound infection |
| **Throat and upper respiratory tract Infections** | J00- nasopharyngitis  J01- sinusitis  J02- streptococcal pharyngitis  J03- tonsillitis  J04- laryngitis  J05- laryngitis and epiglottitis  J06- upper respiratory tract infection  J31- chronic nasopharyngitis  J32- chronic sinusitis  J34.0- nasal abscess  J35.0- chronic tonsillitis  J36- peritonsillar abscess  J37- chronic larynges and laryngotracheitis  J39.0, J39.1- pharyngeal abscess |
| **Ear Infections** | H60, H62.0- otitis externa  H65, H66- otitis media  H67.0- otitis media |
| **Dental/oral Infections** | K02- dental caries  K04.4- apical periodontitis  K04.6, K04.7- dental abscess  K05- gingivitis and periodontal disease  K11.3- salivary gland abscess  K12- stomatitis |
| **Strep A Infections** | A40- streptococcal sepsis  A38- scarlet fever  B95.0- group A strep class other |
| **STI and reproductive tract Infections** | N41.0-3- prostatitis/abscess of prostate  N43.1- infected hydrocele  N45- orchitis and epididimo-orchitis  N48.1- balanitis  N48.2- inflammation of penis  N49- inflamed seminal vesicles  N51- other  N70- salpingitis and oophoritis  N71- infective disease uterus  N72- inflammatory disease of cervix and uteri  N73- other female pelvic inflammatory disease  N74- other female pelvic infection  N75.1 bartholin’s cyst  N764.4 abscess of vulva  N76- inflammation/abscess vulva and vagina  O03.0, O03.5, O05.0, O05.5, O06.0, O06.5, O07.0, O07.5, O04.0, O04.5, O08.0- abortion/ pelvic infection  O23- genitourinary infections in pregnancy  O41.1- amniotic sac infection  O753- infection in labour other  O85, O86- puerperal sepsis  O91- mastitis  O98- other pregnancy/labour/post birth  A50, A51, A52, A53- syphilis  A54- gonorrhoea  A55- chlamydia  A56, A57, A58, A63, A64- other sexually transmitted diseases  A59- trichomoniasis  A60- herpes |
| **Urinary Infections** | N13.6- pyelonephritis  N15.1- perinephric abscess  N30.0- cystitis  N34- urethral syndromes  N39.0- UTI not specified |
| **Bone/joint/connective tissue Infections** | M46.2- osteomyelitis  M46.3, M46.4- discitis  M46.5- infective spondylopathies  M86- osteomyelitis  M00- joint infections  M60.0, M63.0, M63.2- myositis  M65.0, M65.1, M68.0- infective tendonitis  M71.0, M71.1- infected bursitis/abscess |
| **Other Infections** | A41- sepsis  A39- meningococcal disease  G00- bacterial meningitis  G06 - CNS abscess  G07- CNS abscess/granuloma specified elsewhere  H10.0, H10.2, H10.3, H10.9- conjunctivitis  H19.0, H19.3- keratitis  H44.0, H45.1- endophthalmitis  I30- pericarditis  I33, I38, I39- endocarditis  I40, I41- myocarditis  N61- breast inflammation  T82.6, T82.7- cardiac device/valve infection  T835, T836, T845, - device urinary infection  T846, T847- device ortho infection  T857- infection device other  T874- infection amputation stump  A20- plague  A21- tularaemia  A22-anthrax  E32.1- abscess of thymus  I88- lymphadenitis  A17- TB nervous system  N74.0- miscelaneous TB  A23, A24, A25, A26, A27, A28, A30, A31, A32, A33, A34, A35, A36, A37, A38, A42, A43, A44, A480, A483, A484, A488, A49, A65, A66, A67, A68, A69, A70, A71, A74, A75, A77, A78, A79, B96- other infections |
| **Injury** | S00-09- face/head injury  S10-19- neck injury  S20- chest injury  S30-39- injury of abdomen, lower back and pelvis  S40-49- injury of shoulder and upper arm  S50-59 -injury of forearm  S60-69- injury of the wrist and hand  S70-79 -injury of the Hip and thigh  S80-89- injury of the lower leg  S90-99- injury of the ankle and foot  T00-07- injuries involving multiple body regions  T08-09- trunk injury, level unspecified  T10-13- injury of upper limb, level unspecified  T14- injury of unspecified body region  T15-19- foreign body  T20-T31- burns  T33-35- frost bite  T36-65- poisoning  T66- unspecified effects of radiation  T68-69- hypothermia  T70- barotrauma  T71- asphyxiation  T73- affects of deprivation  T74-5- maltreatment syndromes  T79- complications of trauma |

## **Appendix D:**

Hospital admission rates for infections and injury in the first year of life per 100 person-years and associated 95% confidence intervals, by age group at ARF/RHD diagnosis. This data is presented graphically in Figure 1.

|  | 0 to 4 years | 5 to 9 years | 10 to 14 years |
| --- | --- | --- | --- |
| **Any infection** | 72.2 (58.9-85.4) | 77.3 (70.5-84.1) | 86.0 (77.0-95.0) |
| **Specific Infections** |  |  |  |
| Lower Respiratory | 27.9 (19.7-36.1) | 32.5 (28.1-36.9) | 32.4 (26.9-37.9) |
| Skin | 11.4 (6.1-16.7) | 11.4 (8.8-14.0) | 12.8 (9.3-16.3) |
| Upper Respiratory | 7.6 (3.3-11.9) | 7.0 (5.0-9.0) | 7.8 (5.1-10.5) |
| Ear | 5.7 (2.0-9.4) | 5.4 (3.6-7.2) | 8.8 (5.9-11.7) |
| Perinatal | 8.2 (3.7-12.7) | 7.5 (5.4-9.6) | 8.1 (5.3-10.9) |
| Strep A | 1.9 (0.0-4.0) | 0.8 (0.1-1.5) | 0.0 |
| Other | 9.5 (4.7-14.3) | 12.8 (10.0-15.6) | 16.2 (12.3-20.1) |
| **Injury** | 5.1 (1.6-8.6) | 2.2 (1.1-3.3) | 1.2 (0.1-2.3) |
